# Comparison of Brain Age With Standard MRI Assessment for Dementia Risk Stratification in Subjective and Mild Cognitive Impairment

**DOI:** 10.1212/WNL.0000000000218418

**Published:** 2026-08-10

**Authors:** Stefan De Vries, Katalin Farkas, Hanneke F.M. Rhodius-Meester, Argonde Corien Van Harten, Calvin Trieu, Alle Meije Wink, Lyduine E. Collij, Jyrki Lötjönen, Wiesje M. Van Der Flier, Frederik Barkhof, Bas Jasperse, Mara Ten Kate

**Affiliations:** 1 Department of Radiology and Nuclear Medicine, Vrije Universiteit Amsterdam, Amsterdam UMC, location VUmc, the Netherlands;; 2 Amsterdam Neuroscience, Brain Imaging, the Netherlands;; 3 Geriatric Medicine, The Memory Clinic, Oslo University Hospital, Norway;; 4 Amsterdam Neuroscience, Neurodegeneration, the Netherlands;; 5 Alzheimer Center Amsterdam, Neurology, Vrije Universiteit Amsterdam, Amsterdam UMC, location VUmc, the Netherlands;; 6 Combinostics Ltd, Tampere, Finland;; 7 Alzheimer Nederland, Amersfoort, the Netherlands;; 8 Epidemiology and Biostatistics, Vrije Universiteit Amsterdam, Amsterdam UMC, location VUmc, the Netherlands;; 9 Amsterdam Public Health, the Netherlands; and; 10 University College London (UCL), United Kingdom.

## Abstract

**Background and Objectives:**

Early dementia risk-stratification is crucial for timely intervention. Brain-predicted age difference (brain-PAD) derived from structural MRI reflects the gap between computer-estimated age and chronological age and has been linked to clinical progression to dementia. We evaluate whether brain-PAD has prognostic value for progression to dementia in subjective cognitive decline (SCD) and mild cognitive impairment (MCI) and whether brain-PAD provides added value beyond visual rating scales.

**Methods:**

This retrospective cohort study included patients with SCD and MCI with up to 10-year follow-up assessments from the Amsterdam Dementia Cohort and SCIENCe project. Brain-PAD was computed from baseline MRI using 2 pretrained methods. Cox proportional hazards models assessed the association between brain-PAD and risk of progression to dementia, controlling for age, sex, Mini Mental State Examination, and visual rating scales. We tested interactions between brain-PAD and baseline diagnosis to assess stage-specific value and evaluated the added value beyond amyloid status in a biomarker-available subset.

**Results:**

The study included 799 patients (SCD 412, MCI 387; 39.4% female, 63.3 ± 8.0 years). During a median follow-up of 3.2 years, 235 (29.4%) progressed to dementia. Higher brain-PAD was associated with an increased risk of progression to dementia (hazard ratio [HR] 1.04, 95% CI 1.02–1.06) and improved model fit beyond visual ratings (χ^2^ = 12.24, *p* = 0.003). Following a significant interaction between brain-PAD and baseline diagnosis (*p* = 0.013), stratified analyses demonstrated brain-PAD was a stronger predictor in SCD (HR 1.09, 95% CI 1.03–1.15) than in MCI (HR 1.01, 95% CI 0.99–1.04). In SCD, brain-PAD improved model fit beyond visual rating scales (χ^2^ = 10.17, *p* = 0.003) and modestly increased discrimination (C-index +0.02). A data-driven brain-PAD cutoff of −2.6 years corresponded to a high negative predictive value (0.91–0.99) over 2–10 years. Although brain-PAD improved model fit beyond amyloid status (χ^2^ = 6.44, *p* = 0.018), it did not improve discrimination. In MCI, brain-PAD provided no additional value (χ^2^ = 0.88, *p* = 0.351).

**Discussion:**

MRI-derived brain-PAD predicts progression to dementia in memory clinic patients with SCD or MCI. In particular, brain-PAD provided additional predictive value to visual rating scales in patients with SCD.

## Introduction

Brain atrophy patterns visible on MRI are a hallmark of neurodegenerative diseases and strongly associated with cognitive decline.^1^ In clinical practice, atrophy is commonly assessed using visual rating scales.^2,3^ However, their limited ability to detect subtle brain changes limits the use of visual rating scales for dementia risk stratification in early disease stages, clinically presenting as subjective cognitive decline (SCD) or mild cognitive impairment (MCI).^4,5^ Nonetheless, early risk-stratification is critical to enable timely interventions, clinical trial access, and support for patients and their peers through timely education, planning, and resources.^6,7^ This calls for more sensitive MRI markers to detect early-stage pathology, when intervention is most effective.

Brain predicted age difference (brain-PAD) quantifies the difference between chronologic age and computer-predicted age from whole-brain MRI (brain age).^8^ Several methods have been developed to predict brain age, including machine learning algorithms like BrainageR^9^ and deep learning models.^10^ Elevated brain-PAD has been associated with various neurodegenerative diseases^11^ and increased risk of progressing to dementia in MCI, SCD, and cognitively unimpaired individuals.^9,12^ This has led to a growing interest in brain-PAD as a global marker of brain health with potential for identifying at-risk individuals in the earliest disease stages.

Several studies have demonstrated the prognostic value of brain-PAD for progression to dementia in research,^13^ memory clinic,^9^ and community dwelling^12^ cohorts. This effect was consistently found even after controlling for known risk factors, such as baseline cognitive performance,^9^ *APOE-e4* carriership, amyloid status,^14^ and volumetric structural MRI measures.^12^ These findings support the value of brain-PAD, but for implementation in routine clinical practice, it is important to know whether brain-PAD has additional value over commonly used visual rating scales. Moreover, previous studies grouped patients with SCD and MCI, preventing stage-specific comparisons.

In this study, we investigate whether brain-PAD can be used to predict progression to dementia in memory clinic patients presenting with SCD or MCI and whether brain-PAD provides added predictive value beyond visual assessment of brain MRI using visual rating scales.

## Methods

### Patients

All patients who were referred to the Alzheimer Center at Amsterdam UMC and enrolled in the Amsterdam Dementia Cohort (ADC)^1^ and SCIENCe project^15^ between 2002 and 2024 were considered for this study. The Alzheimer Center is a tertiary academic referral center, serving a relatively young patient population. Inclusion criteria were baseline diagnosis of MCI or SCD, the availability of Mini Mental State Examination (MMSE),^16^ and brain MRI at the time of first evaluation. Patients were included when at least one clinical follow-up was performed within 10 years of the initial visit.

At the initial visit, patients underwent a standardized diagnostic workup. This included physical and neurologic examination, a neuropsychological test battery, MRI, electroencephalography, lumbar punction, and routine laboratory testing.^1^ Diagnoses were made in a multidisciplinary consensus meeting. MCI was diagnosed according to the MCI criteria from the National Institute on Aging-Alzheimer's Association,^17^ following earlier use of the Petersen criteria^18^ SCD was diagnosed if the patient presented with cognitive complaints without objective evidence of cognitive dysfunction on clinical and neuropsychological assessments (criteria for MCI, dementia, or any psychiatric disorder not met). Patients were invited for annual standardized follow-up visits, which included medical examination and neuropsychological testing. Follow-up dementia diagnosis was made according to common clinical and research criteria.^19-21^ Time to dementia was defined as the time between baseline visit and time of dementia diagnosis.

Patients with low-quality T1 MRI (n = 8) and those who obtained a dementia diagnosis within 3 months after the screening day (n = 3) were excluded from the study. The final sample consisted of 799 patients (SCD: n = 412, MCI: n = 387). Patients were followed for a median of 3.2 years, ranging from 3 months to 10 years.

Amyloid status was obtained using CSF biomarker analyses and/or amyloid PET. The CSF samples were measured using ELISA, while amyloid-PET scans were visually read by a nuclear medicine physician.^15^ When both PET and CSF were available, we used the PET measure to derive amyloid status. Amyloid status was available for 85.0% (679/799) of the patients. In the SCD group, 26.6% (89/334) were amyloid positive and in the MCI group, 69.0% (238/345).

### Standard Protocol Approvals, Registrations, and Patient Consent

Written informed consent was obtained in accordance with the Declaration of Helsinki of 1975 and the local ethics board of Amsterdam UMC.

### MR Visual Ratings

T1-weighted MR images were visually assessed during the initial evaluation at the memory clinic of Amsterdam UMC by a trained rater, followed by a confirmatory evaluation with an experienced neuroradiologist as part of our clinical routine. Atrophy was evaluated using the medial temporal lobe atrophy (MTA) scale^2^ to assess hippocampal atrophy, Koedam scale for parietal atrophy (PA),^22^ and global cortical atrophy (GCA) scale.^23^ Visual rating scales for MTA and PA were assessed for both hemispheres, with left and right scores averaged to obtain a single value per scale. On fluid-attenuated inversion recovery (FLAIR), white matter hyperintensities were assessed using the Fazekas scale. MRI was obtained across 8 different scanners using a standardized protocol, see eTable 1.

### Brain Age Estimation

Brain age was estimated using the open-source application BrainageR^9^ and the brain age implementation by the regulatory cleared cNeuro cMRI tool (Combinostics, Finland). Both methods have been shown to perform at a level comparable with other brain age estimation approaches in the literature, with a reported mean absolute error (MAE) of 3.93 and 4.40 years for BrainageR and cNeuro, respectively.

The 2 applications predicted brain age in different ways. For BrainageR, the instructions on the associated GitHub page were followed.^24^ The T1-weighted scans were processed with SPM12 to obtain segmentations for gray matter, white matter, and CSF, which were visually inspected for quality.^25^ A predefined principal components analysis rotation was applied, and brain age was then estimated using a Gaussian process regression model.

For cNeuro, the T1-weighted scans were uploaded to the cNeuro platform. Brain age was estimated using a convolutional neural network (CNN) that takes probabilistic tissue segmentations derived from the T1-weighted images as input. The CNN was trained on T1 images from 7,578 patients covering a broad age range. Within the cNeuro pipeline, tissue segmentation, quality control, and brain age estimation were performed automatically.

For both methods, Brain-PAD was computed as the difference between the estimated brain age and chronologic age.

### Statistical Analysis

All statistical analyses were performed in R (v4.3.2). Patients were classified into 2 groups based on their follow-up diagnosis. Patients were designated as “converter” when a dementia diagnosis was made at follow-up (SCD to dementia: n *=* 39, or MCI to dementia, n = 196), and as “nonconverter” when patients remained SCD or MCI (n = 561), including patients who progressed from SCD to MCI without further progression (n = 3).

Baseline characteristics of converters and nonconverters were compared using independent *t* tests for normally distributed continuous variables and χ^2^ tests for categorical variables. For non-normally distributed continuous variables and ordinal variables, such as visual rating scales, Wilcoxon tests were used. Comparisons were subsequently stratified by baseline diagnoses.

### Survival Analysis

Cox proportional hazards regression was used to assess whether baseline brain-PAD predicted conversion to dementia. Follow-up was right censored at 10 years (95th percentile of follow-up duration rounded up) to reduce the influence of outliers. All models included age, sex, and baseline MMSE^16^ as covariates. Interactions between brain-PAD and age or sex were evaluated and included as covariates where significant. To assess the need for subgroup analyses, we tested the interaction between brain-PAD and baseline diagnosis. Following a significant interaction, we performed stratified analyses for SCD and MCI separately.

Cox regression models were developed to evaluate the added predictive value of brain-PAD for progression to dementia. Three models were specified and each was extended with brain-PAD, resulting in 6 models in total. Model 1 included age, sex, and MMSE. Model 2 extended Model 1 with MTA, PA, GCA, and Fazekas. Model 3 extended Model 2 with amyloid status. To assess the incremental value of brain-PAD, each base model was compared with its corresponding brain-PAD variant using likelihood ratio tests. C-index was calculated to demonstrate the discriminative ability of each combination of measures. Continuous net reclassification index (NRI) was calculated 5 years after baseline to indicate the percentage of patients who obtained a more appropriate risk prediction when brain-PAD was included. Afterward, the survival analysis was repeated using brain-PAD measures from the commercially available tool cNeuro.

The proportional hazards assumption was tested for all Cox regression models using Schoenfeld residuals. Multicollinearity among covariates was assessed using variance inflation factor. To account for multiple testing, *p* values for brain-PAD hazard ratios and likelihood ratio tests based on BrainageR were corrected using the false discovery rate method.

### Data-Driven Brain-PAD Cutoff

We aimed to determine the point at which brain-PAD most effectively separates converters from nonconverters within our cohort. For SCD and MCI groups separately, Cox models were fitted with brain-PAD as the sole predictor. Time-dependent ROC curves were computed at 2, 4, 6, 8, and 10 years, and the cutoff maximizing the average Youden index (sensitivity + specificity − 1) across these horizons was selected. To assess the internal stability of these cutoffs, we performed 100 bootstrap iterations. Negative predictive value (NPV) and positive predictive value (PPV) at the final cutoff were calculated for each follow-up horizon.

Patients were dichotomized into “high” and “low” brain-PAD groups using the found cutoffs. The dichotomized brain-PAD variable was subsequently incorporated into multivariable Cox regression models adjusted for age, sex, MMSE, MTA, PA, GCA, and Fazekas. Kaplan-Meier survival curves stratified by brain-PAD group were generated for illustration.

### Data Availability

Anonymized data that support the findings of this study are available on reasonable request from a qualified investigator.

## Results

Baseline demographics are shown in Table 1. During follow-up, 235 (29.4%) individuals converted to dementia (SCD = 39; MCI = 196). Dementia was diagnosed after a median of 3.2 years (interquartile range [IQR] = 4). Individuals who converted to dementia were at baseline older, more often diagnosed with MCI, had lower MMSE-scores, and had higher rates of atrophy on all visual scales compared with those who did not develop dementia. Table 2 summarizes which patients with dementia progressed to.

**Table 1:** Demographic and Clinical Sample Characteristics per Group and Converter Status

Subset	Variable	Total	Converter	Nonconverter	*p* Value
Total	N	799	235	564	
	Baseline diagnosis (MCI), n (%)	387 (48.4)	196 (83.4)	191 (33.9)	<0.001^a^
	Follow-up time, median (IQR)	3.2 (4)	2.9 (2.3)	3.6 (4.2)	<0.001^b^
	Sex (female), n (%)	315 (39.4)	93 (39.6)	222 (39.4)	1.000^a^
	Age, mean (SD)	63.3 (8)	65.5 (6.6)	62.3 (7.9)	<0.001^c^
	MMSE, mean (SD)	27.4 (2)	26.4 (2.4)	27.8 (2)	<0.001^b^
	GCA, median (IQR)	0 (1)	1 (1)	0 (1)	<0.001^b^
	MTA, median (IQR)	0.5 (1)	1 (1.5)	0.5 (1)	<0.001^b^
	Koedam, median (IQR)	1 (1)	1 (0)	1 (1)	<0.001^b^
	Fazekas, median (IQR)	1 (1)	1 (1)	1 (1)	<0.001^b^
	Microbleeds, presence (%)	167 (20.9)	62 (26.4)	105 (18.6)	0.018^a^
	Amyloid+, n (%)	327 (48.2)	175 (83.3)	152 (32.4)	<0.001^a^
SCD	N	412	39	373	
	Follow-up time, median (IQR)	4.4 (5)	3.7 (4.2)	4.4 (4.5)	0.464^b^
	Sex (female), n (%)	172 (41.7)	9 (23.1)	163 (43.7)	0.021^a^
	Age, mean (SD)	61 (8)	65.7 (6.5)	60.5 (7.6)	<0.001^c^
	MMSE, mean (SD)	28.3 (2)	27.4 (1.7)	28.4 (1.5)	0.001^b^
	GCA, median (IQR)	0 (1)	0 (1)	0 (1)	0.004^b^
	MTA, median (IQR)	0 (0)	0 (1)	0 (0.5)	0.074^b^
	Koedam, median (IQR)	0 (1)	1 (1)	0 (1)	0.005^b^
	Fazekas, median (IQR)	1 (1)	1 (1)	1 (1)	0.180^b^
	Microbleeds, presence (%)	70 (17.0)	14 (35.9)	56 (15.0)	0.002^a^
	Amyloid+, n (%)	89 (26.6)	27 (79.4)	62 (20.7)	<0.001^a^
MCI	N	387	196	191	
	Follow-up time, median (IQR)	2.7 (3)	2.7 (2.1)	2.7 (3.7)	0.760^b^
	Sex (female), n (%)	143 (37)	84 (42.9)	59 (30.9)	0.020^a^
	Age, mean (SD)	65.7 (7)	65.5 (6.6)	65.9 (7.4)	0.595^c^
	MMSE, mean (SD)	26.4 (2)	26.2 (2.5)	26.7 (2.3)	0.023^b^
	GCA, median (IQR)	1 (1)	1 (1)	1 (1)	0.077^b^
	MTA, median (IQR)	1 (2)	1 (1)	1 (1)	0.137^b^
	Koedam, median (IQR)	1 (0)	1 (0)	1 (1)	0.061^b^
	Fazekas, median (IQR)	1 (1)	1 (1)	1 (1)	0.702^b^
	Microbleeds, presence (%)	97 (25.1)	48 (24.5)	49 (25.7)	0.883^a^
	Amyloid+, n (%)	238 (69)	148 (84.1)	90 (53.3)	<0.001^a^

Abbreviations: GCA = global cortical atrophy; IQR = interquartile range; MCI = mild cognitive impairment; MMSE = Mini Mental State Exam; MTA = medio temporal atrophy; SCD = subjective cognitive decline.

*p* values were obtained using χ^2^ tests^a^, Wilcox Rank test^b^, and t-tests^c^.

**Table 2:** Number of Patients Progressing to Dementia per Dementia Type

Dementia	SCD	MCI	Total
AD	22	156	178
VaD	2	16	18
DLB	7	10	17
FTD	3	5	8
Dementia other	5	9	14

Abbreviations: AD = Alzheimer disease; DLB = dementia with Lewy bodies; FTD = frontotemporal dementia; MCI = mild cognitive impairment; SCD = subjective cognitive decline; VaD = vascular dementia.

### Brain-PAD Characteristics and MR Correlates

**Figure 1:**
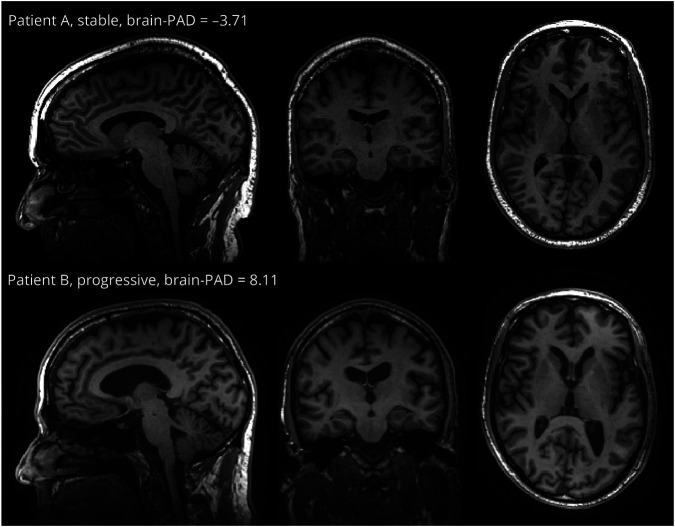
MRI-Scans From Converting and Nonconverting Patients With SCD Matched on MRI-Scanner, Age, Sex, MMSE, and Visual Atrophy Scale Scores brain-PAD = brainpredicted age difference; MMSE = Mini Mental State Examination; SCD = subjective cognitive decline.

Brain age from BrainageR had a MAE of 6.60 (SD = 4.76) and strongly correlated with chronologic age (*r* = 0.73, *p* < 0.001). Brain-PAD was weakly correlated with MTA (*r* = 0.28, *p* < 0.001), PA (*r* = 0.33, *p* < 0.001), GCA (*r* = 0.34, *p* < 0.001), and Fazekas (*r* = 0.23, *p* < 0.001). Representative MRI scans from matched patients illustrating differences between low and high brain-PAD cases are shown in Figure 1.

### Brain-PAD Predicting Progression to Dementia

Brain-PAD was associated with progression to dementia (hazard ratio [HR] 1.06, 95% CI 1.04–1.08, *p* < 0.001), independent of age, sex, and MMSE at baseline. This indicates that for each year the brain appeared older than the chronologic age, the relative risk of future dementia increased by 6%. No significant interactions were observed between brain-PAD and either age or sex. The predictive value of brain-PAD was slightly reduced in models that also included visual rating scales (HR 1.04, 95% CI 1.02–1.06, *p* = 0.003) and amyloid status (HR 1.04, 95% CI 1.01–1.06, *p* = 0.003; Table 3), respectively. Model fit improved in all models when brain-PAD was included, while discriminative ability changed only marginally (ΔC-index < 0.02; Table 3). Continuous NRI showed that adding brain-PAD significantly improved risk stratification over clinical (25% [95% CI 10%–36%]) and visual MRI models (15% [95% CI 1–27]), although this effect was no longer significant after adjustment for amyloid status (13% [95% CI -20%-35%]).

**Table 3:** Independent Hazard Ratios for Brain-PAD as a Predictor of Progression to Dementia Across Clinical Subgroups for Two Brain Age Methods

		BrainageR		cNeuro	
Group	Adjusted for	Brain-PAD HR [95% CI] (per year)	*p* Value	Brain-PAD HR [95% CI] (per year)	*p* Value
Total	1: Clinical	1.06 [1.04–1.08]	<0.001	1.11 [1.09–1.14]	<0.001
	2: + Visual MRI	1.04 [1.02–1.06]	0.003	1.09 [1.06–1.12]	<0.001
	3: + Amyloid	1.04 [1.01–1.06]	0.003	1.08 [1.05–1.11]	<0.001
SCD	1: Clinical	1.09 [1.04–1.15]	0.003	1.15 [1.07–1.24]	<0.001
	2: + Visual MRI	1.09 [1.03–1.15]	0.003	1.17 [1.08–1.26]	<0.001
	3: + Amyloid	1.08 [1.02–1.15]	0.021	1.13 [1.03–1.24]	0.009
MCI	1: Clinical	1.03 [1.00–1.05]	0.03	1.06 [1.03–1.08]	<0.001
	2: + Visual MRI	1.01 [0.99–1.04]	0.351	1.04 [1.01–1.07]	0.007
	3: + amyloid	1.02 [0.99–1.05]	0.178	1.06 [1.02–1.09]	<0.001

Abbreviations: brain-PAD = brain-predicted age difference; GCA = global cortical atrophy; MCI = mild cognitive impairment; MMSE = Mini Mental State Examination; MTA = medial temporal lobe atrophy; PA = parietal atrophy; SCD = subjective cognitive decline.

Adjustment 1 includes age, sex, and MMSE; adjustment 2 includes adjustment 1 plus visual MRI (MTA, PA, GCA, and Fazekas); adjustment 3 includes adjustment 2 plus amyloid status.

A significant interaction was observed between brain-PAD and the baseline diagnosis (HR 1.07, 95% CI 1.01–1.12, *p* = 0.015), revealing that the prognostic value of brain-PAD differed by baseline diagnosis. Stratified analyses showed that in SCD brain-PAD was more strongly associated with progression (HR 1.09, 95% CI 1.04–1.15, *p* = 0.003) than in MCI (HR 1.03, 95% CI 1.00–1.05, *p* = 0.030). When visual rating scales and amyloid status were included, brain-PAD remained a significant predictor in SCD, but the effect was no longer predictive in MCI, see Table 3. In SCD, adding brain-PAD improved model fit and discrimination, as reflected by significant likelihood ratio tests and higher C-index values, whereas no improvement was observed in MCI (Table 4).

**Table 4:** Likelihood Ratio Tests and c-Indices for Models Predicting Progression to Dementia Using Brain-PAD, Visual Ratings, Volumetry, and Amyloid Status in SCD, MCI, and Total Cohorts

		Likelihood ratio test				C-index		
Group	Base model	LL base model	LL base model + brain-PAD	χ^2^	*p* Value FDR adjusted	Base model (SD)	Base model + brain-PAD (SD)	Delta
Total	1: Clinical	−1,349.48	−1,331.41	36.14	<0.001	0.71 (0.02)	0.73 (0.02)	+0.02
	2: + Visual MRI	−1,321.25	−1,315.13	12.24	0.003	0.76 (0.02)	0.76 (0.01)	0.00
	3: + Amyloid	−1,092.20	−1,087.20	10.00	0.003	0.80 (0.02)	0.80 (0.02)	0.00
SCD	1: Clinical	−187.73	−181.98	11.50	0.003	0.75 (0.04)	0.80 (0.04)	+0.05
	2: + Visual MRI	−186.24	−181.15	10.17	0.003	0.77 (0.04)	0.79 (0.04)	+0.02
	3: + Amyloid	−138.20	−134.98	6.44	0.018	0.84 (0.04)	0.84 (0.04)	0.00
MCI	1: Clinical	−986.93	−984.27	5.31	0.029	0.59 (0.02)	0.59 (0.02)	0.00
	2: + Visual MRI	−976.13	−975.69	0.88	0.351	0.63 (0.02)	0.63 (0.02)	0.00
	3: + Amyloid	−836.65	−835.64	2.02	0.178	0.69 (0.02)	0.69 (0.02)	0.00

Abbreviations: brain-PAD = brain-predicted age difference; FDR = false detection rate; GCA = global cortical atrophy; LL = log likelihood; MCI = mild cognitive impairment; MMSE = Mini Mental State Examination; MTA = medial temporal lobe atrophy; PA = parietal atrophy; SCD = subjective cognitive decline.

Model 1 = Sex + age + MMSE; Model 2 = Model 1 + MTA + PA + GCA + Fazekas; model 3 = model 2 + amyloid status.

**Figure 2:**
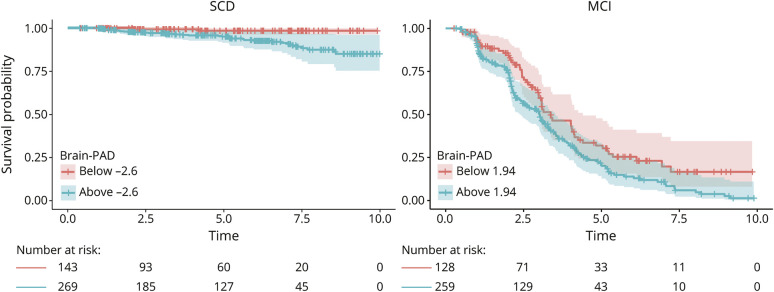
Kaplan–Meier Survival Curves Stratified by the Optimal Brain-PAD Cutoff Found With Youden Index for SCD and MCI brain-PAD = brain-predicted age difference; MCI = mild cognitive impairment; SCD = subjective cognitive decline.

Brain-PAD cutoffs that most effectively separated converters from nonconverters within our cohort were −2.60 years in SCD and 1.94 years in MCI. In SCD, this cutoff showed consistently high negative predictive values across follow-up, indicating strong ability to identify individuals unlikely to progress to dementia (NPV = 0.99 at 2 years; and 0.91 at 10 years), whereas positive predictive value remained low throughout follow-up (PPV = 0.05–0.31). In MCI, predictive performance changed notably over time. At 2 years, the cutoff yielded a high NPV but low PPV (0.89 and 0.20, respectively), which shifted to a low NPV and high PPV at 10 years (0.28, 0.91, respectively). Kaplan-Meier curves illustrate the corresponding risk stratification (Figure 2).

### Replication Using a Different Method for Estimation of Brain Age

Results obtained with cNeuro were mostly consistent with BrainageR. Brain age estimates derived from cNeuro strongly correlated with chronologic age (*r* = 0.79, *p* < 0.001), with a MAE of 5.23 years (SD = 4.01). Replication of the survival analysis using cNeuro-derived brain-PAD estimates yielded similar results as BrainageR, with slightly higher hazard ratios for brain-PAD across models (Table 3). Stratified analyses demonstrated that the predictive value of cNeuro-derived brain-PAD for future dementia was the strongest in SCD, similar to BrainageR. In contrast to BrainageR, cNeuro-derived brain-PAD remained a significant predictor in MCI when visual rating scales and amyloid status were included (Table 3). Model comparison data and C-indices are available in eTable 2.

## Discussion

Using 2 brain age estimation methods, we found that brain-PAD was a robust and independent predictor for progression to dementia in memory clinic patients with SCD and MCI. Each year the brain appeared older than the chronologic age was associated with a 6% increased relative risk of progressing to dementia, independent of age, sex, and baseline MMSE. This decreased to 4% after further adjusting for visual rating scales (MTA, GCA, and PA). Notably, brain-PAD was most informative in patients with SCD, where it was associated with an increased relative risk of 9%. However, in MCI, brain-PAD did not add value beyond visual rating scales.

These findings align with previous studies reporting hazard ratios ranging from 3% to 10% per additional year of brain-PAD. For instance, a study^13^ using ADNI data and focused exclusively on MCI, found a 10% increased relative risk after adjusting for demographic variables. Another study^9^ reported a 3% increase in a memory clinic population, controlling for demographics, MMSE, and normalized brain volume. Research in a community-dwelling sample^12^ observed a 9% increase, even after adjusting for global and regional volumetric measures. Similar to our findings, the relevance of brain-PAD reduced when other measures of atrophy were controlled for. Finally, a study in patients with SCD and MCI^14^ showed that brain-PAD predicted cognitive decline, independent of *APOE-e4*, amyloid-PET status, and bilateral MTA scores. Building on this evidence, we demonstrate that brain-PAD complements standard clinical assessment and, within this context, is particularly valuable in SCD to reassure worried well.

Consistent with previous studies, we confirm that brain-PAD predicts progression to dementia. However, our study extends this evidence by embedding brain-PAD in a setting reflective of clinical practice, where MRI is to date more often interpreted through visual rating scales, rather than volumetrics biomarkers.^3^ Although previous work has convincingly demonstrated the distinct prognostic value of brain-PAD, it has not directly addressed how brain-PAD might complement standard clinical assessment of MRI. Our results show that brain-PAD captures variance not explained by widely used visual rating scales. This supports its potential as a complementary early-stage measure, particularly for patients who may otherwise appear age conform on routine MRI assessment.

Stratified analyses revealed that the prognostic value of brain-PAD varied across disease stages, with the strongest effect in SCD. In this group, brain-PAD provides unique prognostic information not captured by visual rating scales. Integrative MRI-derived markers such as brain-PAD may be better suited to detect the earliest neuroanatomical changes in neurodegenerative disease. This is further supported by studies showing that gray matter organization^26^ and cortical thickness^27^ also provide prognostic value in SCD. In contrast, in MCI, brain-PAD appears to provide little additional predictive value beyond visual ratings, suggesting that at later stages, visual rating scales already capture the majority of risk-relevant structural changes.

To explore the potential utility of brain-PAD for risk stratification, we identified data-driven cutoffs within our cohort. In individuals with SCD, a lower brain-PAD (cutoff of −2.6 years) was characterized by a high negative predictive value that remained stable over a 10-year period. This suggests that a low brain age compared with chronological age is strongly associated with remaining dementia-free. Although this method requires external validation before this can be considered for clinical use, our findings highlight the potential of brain-PAD to serve as a simple and clinically useful rule-out test to reassure patients with SCD at a low risk of decline.

When amyloid status was included in the models, brain-PAD remained a significant predictor in both the full sample and in SCD. However, the minimal change in discriminative ability, as measured by the C-index, indicates that brain-PAD adds little incremental clinical value once amyloid status is known. These findings suggest that brain-PAD may be most informative in settings where amyloid biomarkers are not routinely available, which is often the case in SCD, where biomarker assessment is generally not recommended.^28^

To assess the robustness of our results, we replicated our findings with a fundamentally different brain age estimation method. Similar to BrainageR, cNeuro-derived brain-PAD was associated with progression to dementia and provided unique prognostic information beyond routinely available clinical measures. Furthermore, the effect was strongest in SCD. In contrast to BrainageR, cNeuro-derived brain-PAD remained a significant predictor in MCI. These results indicate that brain-PAD is a robust predictor of dementia progression, while also highlighting that different methods may vary in sensitivity to future cognitive decline. Further research is needed to understand the factors driving these differences between methods.

In addition to differences between models, the MRI scanner itself may bias the brain-PAD estimates.^29^ The use of 8 different MRI scanners in this study introduced scanner-specific offset in brain-PAD estimates. Through sensitivity analysis (eTable 1), we demonstrated that although brain-PAD estimates were biased by MRI scanners, the overall prognostic value remained stable with and without correction. However, this bias has implications for the clinical interpretation of brain-PAD. Specifically, comparing brain-PAD with universal cutoff may be unreliable without calibration.

This study has several strengths. First, it was conducted in a memory clinic cohort with clearly defined and relatively large groups of patients with SCD and MCI. Diagnoses were established through multidisciplinary consensus, including CSF biomarkers or amyloid-PET scan for many patients, improving diagnostic accuracy. In addition, the extensive follow-up time strengthened the assessment of long-term clinical outcomes. Second, we demonstrated the robustness of our findings using 2 independent brain age estimation methods. Both methods are pretrained, improving reproducibility and implementation. Finally, our analyses were designed to reflect real-world clinical workflows, controlling only for routinely available variables such as age, sex, MMSE, and visual rating scales. This approach allowed us to assess the added prognostic value of brain-PAD in a way that aligns with current diagnostic practice.

This study also has limitations to consider. The ADC is primarily focused on younger patients, which may limit the generalizability of our findings to older populations at risk for dementia, who may have more (age-related) atrophy. Similarly, our cohort largely consisted of patients progressing to Alzheimer disease dementia, future research is warranted on the prognostic value of brain-PAD across etiologies. Furthermore, visual rating scales alone do not fully reflect the evaluation of MRI by a trained radiologist. This could be addressed by prospective studies integrating brain-PAD into clinical workflow to examine the added value of brain-PAD more accurately.

In conclusion, we demonstrated that brain-PAD is an independent predictor of progression to dementia in memory clinic patients. It adds prognostic value beyond established visual rating scales, especially in patients with SCD, where conventional MRI assessment often lacks sensitivity. Although further validation is required, our findings highlight the potential of brain-PAD as a sensitive, automated complement to assessment of visual rating scales for identifying individuals at a risk of progressing to dementia.

## Glossary

ADC: Amsterdam Dementia Cohort
brain-PAD: brain-predicted age difference
CNN: convolutional neural network
GCA: global cortical atrophy
HR: hazard ratio
IQR: interquartile range
MAE: mean absolute error
MCI: mild cognitive impairment
MMSE: Mini Mental State Examination
MTA: medial temporal lobe atrophy
NPV: negative predictive value
NRI: net reclassification index
PA: parietal atrophy
PPV: positive predictive value
SCD: subjective cognitive decline
